# Electroacupuncture Restores 5-HT System Deficit in Chronic Mild Stress-Induced Depressed Rats

**DOI:** 10.1155/2016/7950635

**Published:** 2016-11-23

**Authors:** Dongmei Duan, Ya Tu, Xiuyan Yang, Ping Liu

**Affiliations:** ^1^Department of Health Care of South Building, Chinese PLA General Hospital, Beijing 100853, China; ^2^Acupuncture College of Beijing University of Chinese Medicine, Beijing 100029, China; ^3^Beijing University of Chinese Medicine, Beijing 10029, China; ^4^Department of Clinical Pharmacology, Chinese PLA General Hospital, Beijing 100853, China

## Abstract

*Objective.* The current study is designed to investigate the antidepressant efficacy of electroacupuncture (EA) treatment by evaluating its effect on the synthesis, metabolism, reuptake, and receptors of 5-hydroxytryptamine (5-HT), so as to clarify the molecular mechanisms of EA for antidepression.* Materials and Methods.* Solitary combined with the chronic unpredictable mild stress (CUMS) was used to establish the rat model with depression. The depressed rats were supplied with EA treatment for 4 weeks, and the behavior change and the following indices including 5-HT, 5-hydroxyindoleacetic acid (5-HIAA), monoamine oxidase A (MAO-A), tryptophan hydroxylase (TPH), 5-HT transporter (SERT), 5-HT1A, and 5-HT2A in hippocampus and prefrontal cortex were examined.* Results.* EA treatment significantly improved the behavior of rats and increased 5-HT level in hippocampus of depressed rats. Similarly, EA treatment could significantly increase protein and mRNA expression of TPH and 5-HT1A during 5-HT synthesis process in hippocampus of depressed rats. However, EA treatment had no effect on the activity of MAO-A and the expression of SERT protein and mRNA.* Conclusion.* Antidepressant efficacy of EA treatment can be accomplished through enhancing 5-HT synthesis, upregulating 5-HT1A level, and improving 5-HT content in brain and synaptic gaps.

## 1. Introduction

Depressive disorders are prevalent psychiatric disorders with a lifetime incidence of 15–25%. Antidepressant medications have been commonly used in treatment for depression, such as fluoxetine, but almost 30% of patients fail to respond to the treatment [1]. Recently, alternative treatment options became a new focus [2]. As a traditional Chinese medical therapy, acupuncture has been widely used to treat depression [3–8]. Acupuncture stimulation at Neiguan (PC6) point can restore stress-induced decrease in the latency in the open arms produced by CUMS; acupuncture stimulation also tends to restore stress-induced decrease in the sucrose intake [9]. However, the underlying mechanism of acupuncture treatment in depression remains unclear.

The pathophysiology of depression is complex and involves several different signal pathways. Since 1965, the monoamine theory of depression advocates that mental depression is due to the deficiency of monoaminergic activity in brain. In addition, a large number of studies have demonstrated that serotonergic system is intimately linked to stress and anxiety responses and plays a major role in the onset and course of depression [10–12]. 5-HT is one of the ubiquitous molecules as a neurotransmitter or neuromodulator and has been detected outside the central nervous system and confirmed as the same entity with the “clotted blood” vasoconstriction effect in 1952 [13, 14].

Our previous studies have demonstrated that EA has the antidepressant-like effect in chronic unpredictable mild stress (CUMS) rats and depressed patients [15–17], and our preliminary studies showed that EA treatment improved 5-HT level. However, the underlying mechanisms of EA treatment for the regulation of 5-HT level in CUMS rats are still unclear. Meanwhile, the mechanisms for regulating the synthesis, metabolism, and reuptake of 5-HT and its receptor functions are very complicated. The impacts on any one of these factors could affect 5-HT content. In the present study, we investigated the impacts of EA treatment on 5-HT system in CUMS rats, which further confirmed the antidepressant efficacy of EA and explored its possible mechanisms.

## 2. Material and Methods

### 2.1. Animals

Wistar rats (male, age of 6-7 weeks, body weight of 180–200 g, specific pathogen-free grade) were purchased from Beijing Vital River Laboratories (License number SCXK (Jing) 2012-0001). All rats were maintained under a temperature and humidity-conditioned environment with a 12 : 12 h light-dark cycle; all rats were provided with free access to food and water except the period of stress stimuli. The experimental protocols were in accordance with the Guidance Suggestions for the Care and Use of Laboratory Animals issued by the Ministry of Science and Technology of China. The experimental procedures were approved by the Animal Experimentation Ethics Committee of General Hospital of Chinese PLA.

### 2.2. Groups and Treatments

The rats were randomly divided into five groups (ten rats in each group) including no stress stimulation as the normal control group, CUMS group, EA group with EA treatment for CUMS rats prior to 1 h of stress exposure once a day from day 33 to day 59 (Table 1), sham EA group as the negative control group, and fluoxetine treatment group as the positive control with oral administration of fluoxetine (20 mg/kg, Eli Lilly Company, USA) at the same treatment period. Fluoxetine was diluted in distilled water and subjected to oral administration prior to 1 h of stress exposure. A previous work has reported that fluoxetine at the dose of 20 mg/kg has obvious antidepressant efficacy [13].

### 2.3. CUMS Procedure

After 3 days of acclimatization under the housing conditions, the rats except normal control group were subjected to CUMS exposure. The CUMS model was established as previously described with minor modifications [18]. Briefly, stress stimuli includes high-speed agitation for 10 min, deprivation of either food and/or water for 24 h, immobilization for 2 h, continuous illumination for 24 h, cage tilting for 12 h, and forced swimming in ice water for 5 min. These stress stimuli lasted for 4 weeks, and each rat was provided with one type of stress stimuli every day. Finally, the rats subjected to the stress stimuli were housed in individual cages to sustain the depressed state. In contrast, the rats in the negative control group were housed in undisturbed cages (five rats in each cage). In order to ensure the unpredictable stress procedure, the sequence of stress stimuli was changed every week. After the stress stimuli, the CUMS rats were screened by sucrose preference test and open-field test.

### 2.4. EA Treatment

The EA treatment was similar as previously described [19]. EA treatment at Baihui (GV 20, the center of parietal bone) and Yintang (EX-HN3, the middle point of the line between the brow bones) points was conducted for depressed model rats for 28 days (from day 33 to day 59). The acupuncture needles (Hwato Brand, number 30, 0.5 cun; Suzhou Medical Instrument Factory, Suzhou, Jiangsu, China) were pricked into the skin at Baihui and Yintang at a depth of 2 mm. A forward oblique needling was required for Baihui, while an upward oblique needling was required for Yintang. EA treatment was provided with a cluster-shaped wave at a frequency of 2 Hz and an intensity of 1 mA. The stimulation level was minimized to avoid struggling or vocalization of the rats. The rats were subjected to EA treatment once a day for 20 min during the 4-week treatment period. For sham EA treatment group, acupuncture needles were shallowly inserted into the acupoints such as EX-HN3 and GV20 without electrostimulation.

### 2.5. Behavior Tests

The sucrose intake was assessed using a previously described method with minor modifications [9, 20]. Briefly, the rats were accustomed to drink 5% sucrose solution for 24 h without any water prior to tests. On the last stressed day, the rats were subjected to water deprivation for 24 h. Subsequently, the rats were given a 1 h window for sucrose test (between 14:00 and 15:00 h). In order to determine the sucrose preference, preweighed bottles of sucrose solution and water were reweighed after the test. All tests were carried out in the home cage to minimize extraneous novelty and disturbance. The sucrose preference was calculated as sucrose intake/(sucrose intake + water intake). Anhedonia was defined as a reduction in sucrose preference relative to baseline levels. The open-field test was also conducted at the end of the experiment. The apparatus consisted of a square arena with the dimension of 80 cm × 80 cm and walls of 40 cm in height were divided into equal squares with 25 cm × 25 cm in dimension in the floor of the arena. A single rat was gently placed in the center of the floor and provided with 5 min for finding the arena. The crossing pieces (defined as at least three paws in a square) and the rearing times (defined as the rat standing upright on its hind legs) were manually and blindly counted by two investigators. In addition, body weights of the rats were determined on day 33 and day 59 of the experiment, respectively. The forced swimming test was conducted in an open cylindrical container (25 cm in diameter and 50 cm in height) containing freshwater at 25 ± 2°C to a depth of body height +5 cm for 13 min. The activity of rats was recorded with a video camera, and immobilization time for 10 min after excluding the first 3 min from 13 min recording duration was measured. The immobilization state is defined as a state in which the rat suspends the movements and accomplishes the movements of the limbs necessary to maintain its balance.

### 2.6. Enzyme-Linked Immunosorbent Assay

Immediately after the behavior tests, all rats were decapitated after anesthesia and the whole brain was quickly removed. Sequentially, prefrontal cortex and hippocampus were isolated and then frozen in liquid nitrogen and stored at −80°C for future analysis. The levels of 5-HT and 5-HIAA in prefrontal cortex and hippocampus were measured using a paired antibody quantitative ELISA kit according to the manufacturer's instructions (ADL Inc., USA). 20 mg prefrontal cortex or hippocampus tissue was added to 1 mL homogenate and homogenated on ice. Dispensed antigen standards (0, 10, 20, 40, 80, and 160 ng/mL) and samples (5 times dilution) were added to each well of 96-well plates precoated with primary antibodies; then the plates were incubated at 37°C for 30 min. After washing the plates and adding biotin-conjugated reagent and enzyme-conjugated reagent to each well, the plates were incubated at 37°C for 30 min again. Then the plates were rinsed with distilled water 5 times and measured under 450 nm within 15 min using a microtiter plate reader (Perkin Elmer, USA). Calculating the sample's concentration on the basis of the standard curve, all data were expressed in ng/mL [18].

### 2.7. Luminescence Assay

Hippocampus was collected on ice plate and weighed; and mitochondrial fraction was prepared using the method previously described by Xia et al. [21]. Briefly, the hippocampus tissue was washed in cold sodium phosphate buffer (PBS; 10 mM, pH 7.4, containing 250 mM sucrose) at 9-fold volume and then resuspended at 100 mg/mL in ice-cold homogenization buffer and homogenized by hand using a small glass homogenizer. Tissue homogenates were then centrifuged at 800 ×g for 5 min at 4°C twice. The supernatant was transferred to a new tube and centrifuged at 12000 ×g for 10 min at 4°C. The pellets were resuspended in the same buffer. Protein concentration was estimated by the BCA method using BSA as the standard. MAO-A activities were measured using a MAO-Glo kit (Promega, USA) according to the manufacturer's instructions. Briefly, an opaque white 96-well plate was prepared and 25 *μ*L of 80 *μ*M MAO-A substrate solution was added to each well. Then the reaction was initiated by adding 25 *μ*L of mitochondrial fraction solution, and the plate was incubated at room temperature for 1 h. The reaction was terminated by adding 50 *μ*L of reconstituted luciferin detection reagent and mixed briefly. Finally, the plate was incubated at room temperature for 20 min, and the luminescence was measured by the luminescence detector (PE, USA).

### 2.8. Western Blotting

For western blot analysis [22], prefrontal cortex and hippocampus were lysed in RIPA buffer (150 mM NaCl, 0.5% deoxycholate, 0.1% sodium dodecyl sulfate (SDS), 50 mM Tris, 1 mg/mL leupeptin, 1 mM dithiothreitol (DTT), 2 mM sodium orthovanadate, 1 mg/mL phenylmethylsulfonyl fluoride (PMSF), 1 mg/mL trypsin inhibitor, and 10 mM aprotinin), schizolysised for 20 min on the ice, and then centrifuged at 12000 ×g for 10 min at 4°C. Finally, the supernatant was transferred to a new tube. The protein samples were heated at 95°C for 8 min and then loaded on a 12% SDS-polyacrylamide gel for electrophoresis and transferred to nitrocellulose membrane. The membrane was blocked 2 h in TBS-T buffer (25 mM Tris, 140 mM NaCl, 27 mM KCl, and 0.02% Tween 20) containing 5% BSA. The target protein was probed with monoclonal anti-5-HT1A, anti-5-HT2A, anti-SERT, or anti-TPH (BioWorld, USA) at 4°C overnight. After washing with TBS-T buffer three times, the membrane was incubated with the appropriate HRP-labeled secondary antibody at room temperature for 1 h, washed three times (5 min each time) in TBS-T buffer, and then developed by ECL system according to the manufacturer's instructions (American, UVP).

### 2.9. Analysis of mRNAs by Real-Time RT-PCR

The real-time RT-PCR was used to evaluate mRNA expression of 5-HT1A, 5-HT2A, TPH, or SERT [23]. Total mRNA was isolated from tissues by the acidic guanidinium thiocyanate method and quantified by spectrophotometry. First-strand cDNA was synthesized from 1 *μ*g of each mRNA sample with random primer and reverse transcription kit (TOYOBO, Japan). PCR reaction was conducted using rat-specific forward primer (5′-TAAATACTGGGCCAGGAGAGG-3′) and reversed primer (5′-GAAGTGTCTTTGCCGCTTCTC-3′) for TPH; forward primer (5′-GTACCACCGAAACGGGTGCA-3′) and reversed primer (5′-TGGTGGATCTGCAGGACATG-3′) for SERT; forward primer (5′-CTGGAATGTAAAGACGCCTG-3′) and reversed primer (5′-CCTTTCCTTCTCACCTCTCT-3′) for 5-HT1A; and forward primer (5′-GGCATAATCAAGAAGCATCA-3′) and reversed primer (5′-TTGTAGCAGATGAAGTGTGG-3′) for 5-HT2A. In order to normalize TPH, SERT, 5-HT1A, and 5-HT2A data for quantitative analysis, we performed RT-PCR on the same cDNA sample using forward primer (5′-TACCCACGGCAAGTTCAACG-3′) and reversed primer (5′-CACCAGCATCACCCCATTTG-3′) for GAPDH as the internal reference. SYBR Green real-time PCR Master Mix (TOYOBO, Japan) was used for real-time PCR to evaluate the abundance of PCR products. Relative expression of mRNA was calculated using the 2^−Δ(ΔCT)^ method, with normalized gene against the internal endogenous GAPDH reference gene for the same sample. All data were expressed as the folds of relative change.

### 2.10. Statistical Analysis

All data were expressed as mean ± standard error (M ± SE). Difference in mean value between groups was analyzed by a one-way analysis of variance (ANOVA) followed by Dunnett's test in order to evaluate the intergroup difference. The statistically significant difference was considered at *P* < 0.05.

## 3. Results

### 3.1. EA Treatment Improves Behavioral Indices

**Figure 1:**
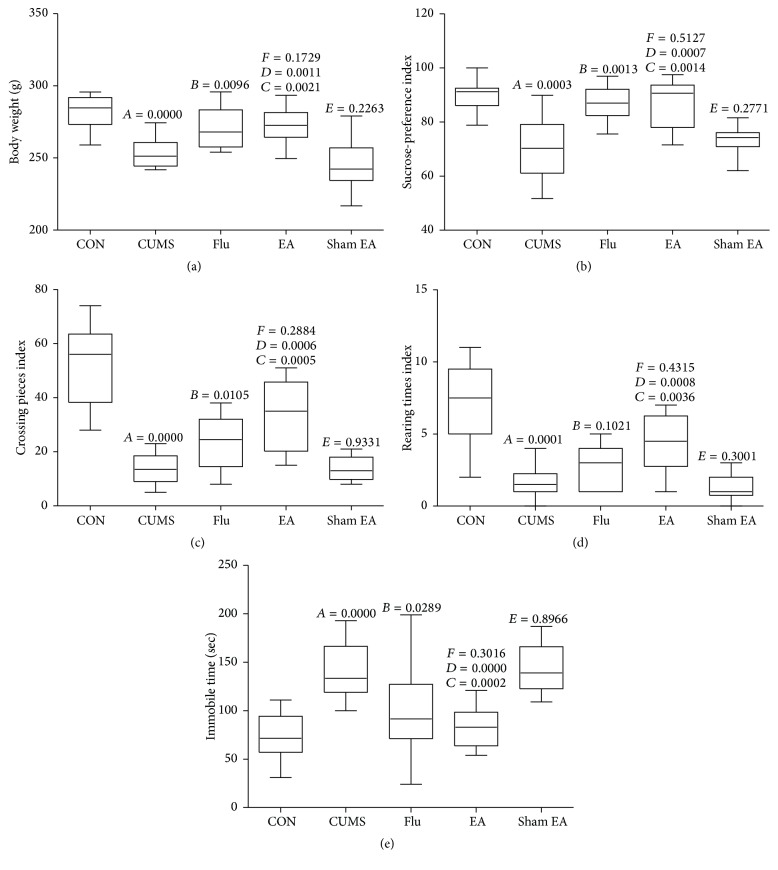
Body weight (a), sucrose preference (b), crossing pieces (c), rearing times (d), and immobile time (e) in CUMS rats with each treatment. CON: normal control group without stress stimuli and any treatments; CUMS: CUMS group exposed to stress stimuli for 8 weeks without any treatments; Flu: CUMS + fluoxetine group exposed to stress stimuli for 8 weeks and fluoxetine treatment at the dosage of 20 mg/kg from the 5th to the 8th week; EA: CUMS + EA group exposed to stress stimuli for 8 weeks and EA treatment (points at Baihui and Yintang) from the 5th to the 8th week; sham EA: CUMS + nonacupoint group exposed to stress stimuli for 8 weeks and without EA treatment (points at EX-HN3 and GV20 without electrostimulator) from the 5th to the 8th week;* A*, CUMS compared with CON;* B*, Flu compared with CUMS;* C*, EA compared with CUMS;* D*, EA compared with sham EA;* E*, sham EA compared with CUMS.

Figure 1 shows the effects of EA treatment on body weight, sucrose preference index, open-field test, and immobilization time. After stress stimulation for 4 weeks, compared with the normal control group, the rats from CUMS group revealed lower crossing number, rearing number, sucrose preference index and body weight, and higher immobilization time (*P* < 0.01). EA treatment could significantly increase crossing number, rearing time, sucrose preference index, and body weight and decrease immobilization time when compared with the CUMS group and sham EA group (*P* < 0.05). Similarly, an increase in crossing number, sucrose preference index, and body weight and a decrease in immobilization time in the fluoxetine treatment group were observed (*P* < 0.05 versus the CUMS group). However, no significant difference between sham EA group and CUMS group was observed (Figure 1).

### 3.2. Determination of 5-HT Levels in Hippocampus and Prefrontal Cortex

**Figure 2:**
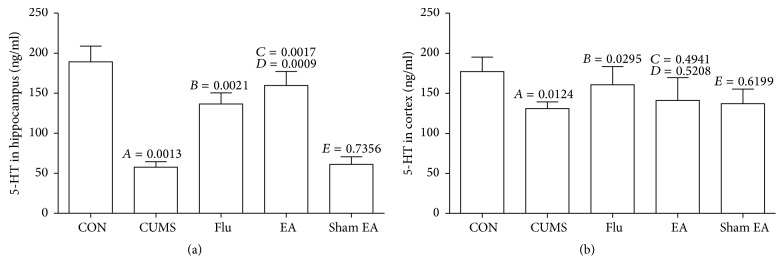
The changes of 5-HT levels in hippocampus and cortex of CUMS rats treated with EA at Baihui and Yintang points. The expression levels of 5-HT in rats were detected by ELISA. (a) 5-HT levels in hippocampus. (b) 5-HT levels in cortex. The results were from the independent experiments in triplicate.* A*, CUMS compared with CON;* B*, Flu compared with CUMS;* C*, EA compared with CUMS;* D*, EA compared with sham EA;* E*, sham EA compared with CUMS.

The effects of EA treatment on 5-HT levels are summarized in Figure 2. In hippocampus and prefrontal cortex, CUMS significantly reduced 5-HT levels (*P* < 0.01 versus normal control group). EA treatment for 4 weeks could rescue the reduction of 5-HT in hippocampus caused by CUMS (*P* < 0.01), and the similar tendency as fluoxetine treatment was observed, but EA treatment had no effect on 5-HT level in prefrontal cortex tissue.

### 3.3. Effect of EA Treatment on Metabolism of 5-HT

**Figure 3:**
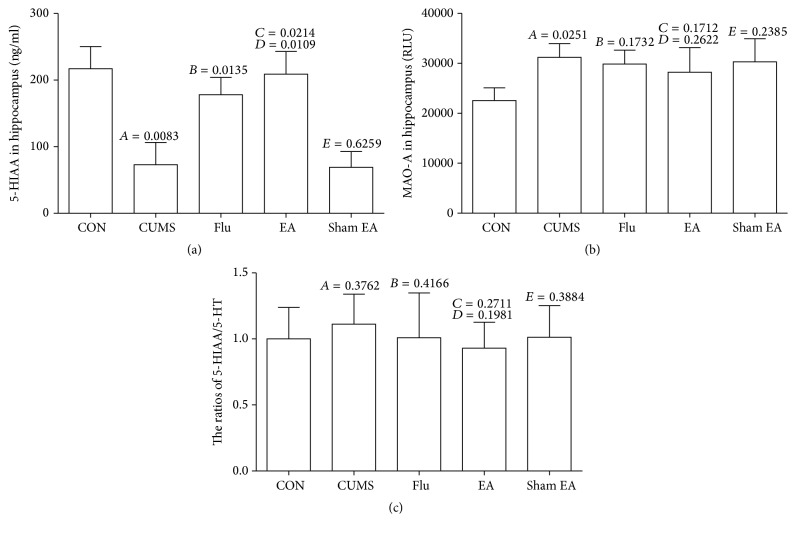
The changes of 5-HIAA level and MAO-A activity in hippocampus of CUMS rats treated with EA. (a) 5-HIAA levels in hippocampus, (b) MAO-A activities in hippocampus, and (c) 5-HIAA/5-HT ratios in hippocampus. These results were from the independent experiments in triplicate.* A*, CUMS compared with CON;* B*, Flu compared with CUMS;* C*, EA compared with CUMS;* D*, EA compared with sham EA;* E*, sham EA compared with CUMS.

We suppose that the elevated 5-HT level from EA treatment may be due to the inhibition of 5-HT metabolism. Then, we detected 5-HIAA level and MAO-A activity in hippocampus of CUMS rats after EA treatment for 4 weeks. In CUMS rats, 5-HIAA content was lower than that in rats from the normal control group, but EA treatment resulted in a significant increase of 5-HIAA level (Figure 3(a)). The activity of MAO-A in hippocampus from CUMS rats was determined by luciferin. As shown in Figure 3(b), the CUMS rats without EA treatment revealed an obvious increase in MAO-A activity when compared with the rats from the normal control group. EA treatment for 4 weeks had no impact on MAO-A activity when compared with CUMS and sham EA treatment. As shown in Figure 3(c), the ratio of 5-HIAA/5-HT in hippocampus was stable so that the change in 5-HIAA might be evoked by the fluctuation of 5-HT.

### 3.4. Effect of EA Treatment on Synthesis and Reuptake of 5-HT and Its Receptor Expression

**Figure 4:**
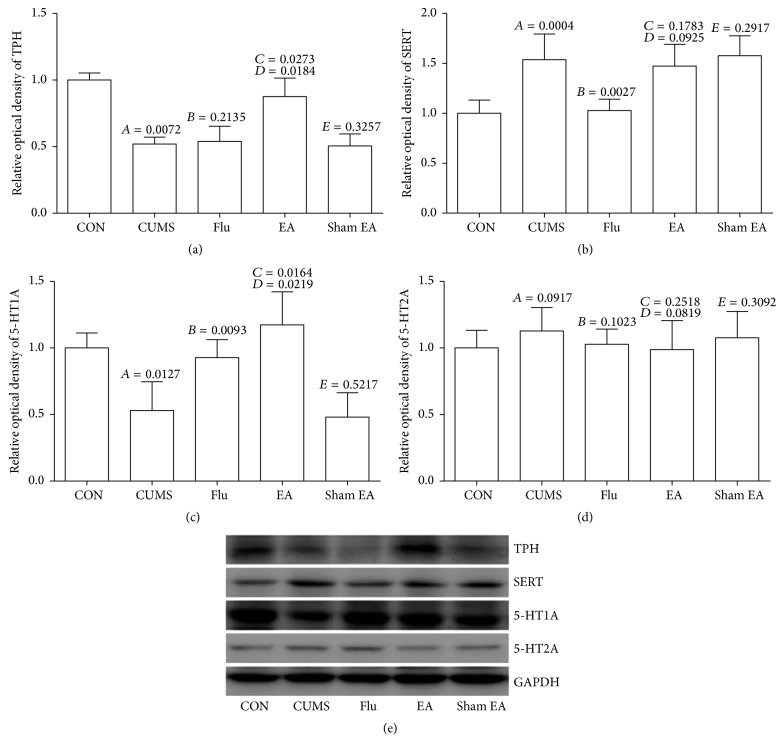
Effect of EA treatment on protein expression of TPH, SERT, 5-HT1A, and 5-HT2A in CUMS rats. The protein expression was detected by western blot. These results were from the independent experiments in triplicate.* A*, CUMS compared with CON;* B*, Flu compared with CUMS;* C*, EA compared with CUMS;* D*, EA compared with sham EA;* E*, sham EA compared with CUMS.

Based on the evaluation by western blotting, the levels of TPH and 5-HT1A proteins in hippocampus revealed a significant decrease and the level of SERT exhibited an obvious increase after chronic mild stress stimulation for 4 weeks, while EA treatment could result in an obvious upregulation of TPH and 5-HT1A when compared with CUMS model rats (Figures 4(a) and 4(c)) but had no effect on SERT expression (Figure 4(b)). On the other hand, 5-HT2A did not exhibit an obvious change after CUMS and EA treatment (Figure 4(d)).

### 3.5. Effect of EA Treatment on mRNAs Expression of 5-HT Synthesis, Reuptake, and Receptor

**Figure 5:**
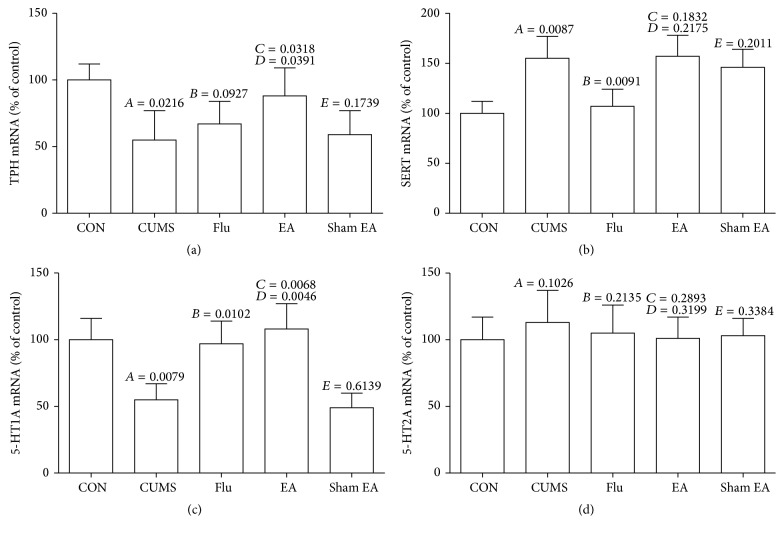
Effect of EA treatment on mRNA expression of TPH, SERT, 5-HT1A, and 5-HT2A in CUMS rats. The expression of TPH, SERT, 5-HT1A, and 5-HT2A at mRNA level was detected by qRT-PCR. These results were from the independent experiments in triplicate.* A*, CUMS compared with CON;* B*, Flu compared with CUMS;* C*, EA compared with CUMS;* D*, EA compared with sham EA;* E*, sham EA compared with CUMS.

Total RNAs were extracted and relative mRNA amounts were measured by real-time PCR after the normalization using GAPDH mRNA as the reference. As shown in Figure 5, TPH and 5-HT1A mRNA levels were significantly upregulated after EA treatment when compared with that in CUMS rats (*P* < 0.01), but no significant difference was observed between CUMS rats with and without fluoxetine treatment in TPH level (Figures 5(a) and 5(c)). SERT mRNA expression was strongly downregulated in fluoxetine treatment groups compared to CUMS control (Figure 5(b)) (*P* < 0.01), but there was no change in EA treatment group. The expression of 5-HT2A mRNA did not reveal an obvious change after CUMS and fluoxetine or EA treatment (Figure 5(d)).

## 4. Discussion

The pathophysiology of depression is complex and involves several different signal pathways. 5-HT, as a neurotransmitter, is confirmed to be involved in depression. In the present study, we detected the monoamine level in brain of CUMS rats after 4-week EA treatment. Two brain regions including frontal cortex and hippocampus involved in emotional, motivational, and mnemonic processes associated with depression were explored.

Abnormal 5-HT system could be due to one or more factors, including 5-HT synthesis, reuptake, or metabolism or its receptors. During the biosynthesis process of 5-HT, tryptophan, as the precursor, can generate 5-hydroxy tryptophan (5-HTP) under the catalysis of TPH, and then 5-HTP can generate 5-HT after decarboxylization by decarboxylase AADC; here TPH is playing as a rate-limiting enzyme. Then 5-HT released into synaptic cleft can bind to the 5-HT1A receptor in postsynaptic membrane for the maintenance and extension of 5-HT energy transfer function. 5-HT in the synaptic cleft is primarily removed through a specific protein such as 5-HT transporter or serotonin transporter in the nerve terminal, namely, SERT [24]. Fluoxetine is a selective serotonin reuptake inhibitor (SSRI), which plays an antidepressive effect by inhibiting the activity of SERT, increasing 5-HT concentration in synaptic cleft. At last, 5-HT is decomposed by MAO in mitochondria. MAO is classified into type A and type B according to their sensitivity towards specificity substrates and acetylenic inhibitors. MAO-A shows a higher affinity for 5-HT and NE [25]. Increased content or activity of MAO often leads to chronic degenerative diseases of the nervous system, and therefore the inhibition of MAO is still the major treatment strategy of depression although strong side effects were observed [26].

In the present study, we detected the effect of EA treatment on CUMS rats to predict its antidepressant efficacy by evaluating the behavioral parameters of CUMS rats. More importantly, EA treatment could result in the improvement of behavior indices and the amelioration of deficient 5-HT system in hippocampus induced by CUMS, indicating remarkable serotonergic activation in brain. 5-HIAA is the catabolic metabolite of 5-HT under the catalysis of MAO-A. In order to verify whether EA treatment has an inhibitory effect on monoamine oxidase and EA is a potent MAO-A inhibitor, we have examined the activity of MAO-A and 5-HIAA in mitochondrial fraction of hippocampus from rats subjected to EA treatment. The results indicated that EA treatment did not inhibit MAO-A activity after CUMS exposure. Thus, the enhanced activity of 5-HT following EA treatment was not correlated with the inhibition of MAO-A. The cheese effect and dietary restriction imposed to those receiving MAO inhibitors were the particularly troublesome aspects of early antidepressants. Tyramine is a substrate for both MAO-A and MAO-B. Nonselective MAO inhibitors can block the first-pass metabolism of exogenous tyramine, thus increasing the risk for the stress response [27–32]. In addition, EA treatment did not reveal a significant inhibition of MAO-A in hippocampus from CUMS rats, indicating that the side effects of EA treatment should be weak.

EA treatment could upregulate 5-HT in hippocampus of CUMS rats. Whether EA treatment can increase the synthesis or reuptake of 5-HT or upregulate its receptor is still unclear. In the present study, we first examined the expression of TPH protein and mRNA, and EA treatment of CUMS rats for 4 weeks could result in a concomitant increase in TPH protein level in hippocampus, thus increasing the synthesis of 5-HT and leading to the enhancement in 5-HT level in depressed rats induced by CUMS. The TPH mRNA was also upregulated in hippocampus. During exploring the effect of EA treatment on 5-HT reuptake, CUMS can induce the protein and mRNA expression of SERT in hippocampus, a statistically significant suppression on the expression of SERT protein and mRNA in hippocampus after fluoxetine treatment is observed, but no impact of EA treatment on SERT is observed. At the same time, EA treatment could upregulate 5-HT1A level in hippocampus of CUMS rats and increase the expression of 5-HT1A mRNA. However, there was no significant difference of 5-HT2A between CUMS group and normal control group and also between EA treatment group and CUMS group.

Based on these findings, the antidepressant efficacy of EA treatment could be accomplished through upregulating 5-HT level, promoting the synthesis of 5-HT and enhancing 5-HT1A level in CUMS rats. As it is well known, monoamine oxidase inhibitor has an antidepressant effect, but its side effects are also obvious, which leads to the limitations of its application as the drug or the diet. EA treatment has no effect on the activity of monoamine oxidase. The auxoaction of EA treatment on 5-HT biosynthesis and 5-HT1A is obvious, but EA treatment cannot significantly inhibit the reuptake of 5-HT like fluoxetine in depressed rats. Therefore, EA treatment can become an antidepressant strategy with the characteristics of innovative mechanisms and high safety, which will provide an important evidence for its clinical application. Also, we argue that the complementary antidepressant mechanism of EA and fluoxetine may provide a certain experimental basis for the combined application of the two treatments, which may have a better antidepressant effect.

## 5. Conclusions

EA treatment can improve the depressed behavior through restoring 5-HT system deficit in chronic mild stress-induced depressed rats. The distinct stimulation of EA treatment on 5-HT is due to the increased 5-HT synthesis and upregulated 5-HT1A receptor, thus finally increasing 5-HT content in brain.

## Figures and Tables

**Table 1:** Schedule for animal experiments.

	CON	CUMS	Flu	EA	Sham EA
D1–D3	Acclimatization				
D4–D31	Normal feeding	CUMS exposure			
D32		Behavior tests and grouping			
D33–D59		No treatment	Flu treatment	EA treatment	Sham EA
D60–D62	Behavior tests				
D63	Collected tissues				
